# Comparative Wing-Scale Ultrastructure Reveals Distinct Structural Modifications Associated with Transparent Wing Regions in Two Moth Species (Lepidoptera)

**DOI:** 10.3390/insects17100999

**Published:** 2026-09-27

**Authors:** Senlin Li, Haifeng Zhou, Zixuan Mu, Shujie Zhang, Yu Liang, Qing Zhang, Kang Lou

**Affiliations:** College of Life Sciences, Zhengzhou University, Zhengzhou 450001, China; senlin_li@zzu.edu.cn (S.L.); zhouhf1025@163.com (H.Z.); m18856798141@163.com (Z.M.); zhangshujie@zzu.edu.cn (S.Z.); liangyyy@gs.zzu.edu.cn (Y.L.); 17837402061@163.com (Q.Z.)

**Keywords:** Lepidoptera, wing transparency, wing scales, ultrastructure, morphological diversity, scanning electron microscopy

## Abstract

Transparent wings are a striking morphological feature in Lepidoptera, yet the scale modifications associated with transparency remain insufficiently understood in many moth groups. In this study, we qualitatively examined the ultrastructure of scales from transparent and opaque wing regions in two moth species, *Amata emma* (Butler) (Erebidae) and *Thyrassia penangae* (Moore) (Zygaenidae), using scanning electron microscopy (SEM). In the examined transparent regions of both specimens, substantial areas of the wing membrane remained exposed between scales, but the scale morphologies differed markedly. In *A. emma*, transparent-region scales were elongated, narrow, and needle-like, whereas those of *T. penangae* projected from the wing membrane and exhibited a distinctive bud-like morphology with folded distal lamellar structures. Opaque-region scales in both specimens were broad, leaf-shaped, and arranged in an overlapping pattern, with conspicuous perforations between longitudinal ridges. Longitudinal SEM observations further revealed a shared hollow internal architecture consisting of upper and lower laminae interconnected by trabeculae, although trabecular morphology differed between scales from the transparent and opaque wing regions. These observations show that macroscopically similar transparent wing regions in the examined specimens can be associated with distinct scale-level structural modifications and highlight the diversity of scale architectures associated with wing transparency in Lepidoptera.

## Simple Summary

Transparent wings are a fascinating feature found in many butterflies and moths. The tiny scales covering the wing surface can influence how much of the wing membrane remains exposed and may therefore be associated with wing transparency. In this study, we examined one specimen from each of two moth species with transparent wing regions and compared the scale structures in transparent and neighboring opaque regions of the forewing. In the examined specimens, the transparent regions showed greater exposure of the wing membrane between scales, but the scales differed in shape and arrangement between the two moths. These observations show that similar transparent wing appearances can be associated with distinct scale configurations. Because only one specimen of each species was examined, the findings describe the specimens studied rather than species-wide patterns. This study provides a morphological basis for future investigations of how different scale structures may contribute to wing transparency.

## 1. Introduction

Wing scales are among the most distinctive morphological features of Lepidoptera and play crucial roles in wing appearance, optical performance, and functional adaptations [1,2,3,4,5,6,7]. Their hierarchical ultrastructure, including surface ornamentation and internal architectures formed by lamellae and supporting elements, governs the interaction between lepidopteran wings and light, and has inspired a wide range of biomimetic applications. Consequently, previous studies have largely focused on the optical mechanisms and developmental processes underlying scale ultrastructure [8,9,10]. However, comparatively little attention has been paid to comparative ultrastructural analyses of wing-scale modifications associated with transparent wing regions in moths [5,11]. Documenting such modifications provides an important basis for understanding the structural basis of transparent wing phenotypes in Lepidoptera.

Transparent wing regions represent one of the most striking morphological features in Lepidoptera [12,13,14]. Unlike opaque wing regions, transparent areas are often associated with reduced pigmentation, decreased scale coverage, or modified scale morphology that alters light transmission [11]. Previous studies have primarily investigated transparent wings in butterflies, focusing on optical properties, developmental mechanisms, and ecological significance [7,13,15,16,17]. In contrast, transparent-winged moths remain comparatively less studied, despite exhibiting diverse transparent window patterns and scale modifications [11,18,19]. Previous studies have further demonstrated that wing transparency in Lepidoptera can arise through diverse structural modifications, including changes in scale morphology, density, orientation, and coverage [5,11,19]. Understanding how different moth species achieve similar transparent wing phenotypes through such scale-level modifications may therefore provide new insights into the structural characteristics associated with transparent wing phenotypes in Lepidoptera.

In this study, we selected two transparent-winged moth species, *Amata emma* (Butler) (Erebidae) and *Thyrassia penangae* Moore (Zygaenidae), which possess conspicuous transparent wing windows and occur on the same larval host plant, *Cayratia japonica* (Thunb.) [20,21,22]. Although these species belong to different families, both exhibit similar transparent wing phenotypes, making them a suitable system for investigating the structural characteristics associated with transparent wing phenotypes across distantly related moth taxa (Figure 1). However, whether their similar transparent phenotypes are achieved through comparable or distinct modifications in wing-scale morphology and ultrastructure remains unknown.

Using scanning electron microscopy (SEM) and comparative morphological analyses, we aimed to (1) document scale morphology and ultrastructure in transparent and opaque wing regions of each species; (2) compare the observed scale organization, surface microstructure, and internal architecture between the two species; and (3) examine whether their similar transparent wing phenotypes are accompanied by similar or distinct scale-level structural modifications.

## 2. Materials and Methods

### 2.1. Specimen Collection and Identification

Adult specimens of *A. emma* and *T. penangae* used for species identification and specimen selection were obtained from the insect collection of Zhengzhou University, Zhengzhou, Henan, China. For each species, two adult specimens were available for morphological identification: two males of *A*. *emma* and one male and one female of *T*. *penangae*. The *A*. *emma* specimens were collected in Yuntaishan Town, Xiuwu County, Jiaozuo City, Henan Province, China (35.43° N, 113.38° E; 389 m a.s.l.) on 13 June 2023, whereas the *T*. *penangae* specimens were collected in Jigongmiao Village, Zhengzhou City, Henan Province, China (34.86° N, 113.53° E; 87 m a.s.l.) on 3 June 2024. All specimens were collected using an insect net by Kang Lou. Species identification was initially based on external morphological characters and was further confirmed by dissection and examination of the male genitalia following the diagnostic descriptions of Wu [22]. The morphology of the male genitalia and aedeagus of the examined specimens was consistent with the diagnostic characteristics of *A. emma* and *T. penangae*, respectively. Representative images of the dissected male genitalia and aedeagus are provided in Figure S1. Following species identification, one adult specimen of each species was selected for SEM examination: one male *A. emma* and one female *T. penangae*.

### 2.2. Scanning Electron Microscopy (SEM)

For each SEM specimen, one forewing was examined using a ZEISS Smartzoom 5 digital 3D microscope (Carl Zeiss Microscopy GmbH, Oberkochen, Germany) to characterize wing morphology and identify the transparent and opaque regions. One transparent region and one adjacent opaque region on the examined forewing were then selected for subsequent ultrastructural observation by SEM. The specific locations of the sampled transparent and adjacent opaque regions are indicated in Figure 2B for *A. emma* and Figure 3B for *T. penangae*. Red boxes labeled “T” indicate the sampled transparent regions, whereas blue boxes labeled “O” indicate the sampled opaque, dark-scaled regions.

Because the overall configuration of the transparent wing windows differed between *A*. *emma* and *T*. *penangae*, strictly corresponding anatomical sampling sites could not be defined between the two examined specimens. Therefore, representative transparent and opaque regions were selected within each specimen to document and compare the scale morphology and ultrastructure associated with these regions.

Individual scales were removed from the selected transparent and adjacent opaque regions of each specimen for SEM preparation. Multiple scales within the sampled regions were examined at different magnifications to document scale morphology, surface structures, and internal architecture, and representative micrographs were selected for presentation. These individual scales and SEM fields constituted subsamples of the same specimen and were not treated as independent biological replicates. For examination of the internal architecture, individual scales were sectioned longitudinally along their longitudinal axis, and the exposed internal surfaces were observed by SEM.

The prepared scale samples were rinsed three times for 15 min each in a 0.1 molar phosphate buffer (pH 7.2), followed by a 30-min fixation in a fixative solution consisting of 2.5% glutaraldehyde (Sangon Biotech (Shanghai) Co., Ltd., Shanghai, China) in 0.1 molar phosphate buffer (pH 7.2) at 4 °C. Samples were dehydrated through a graded ethanol (Sangon Biotech (Shanghai) Co., Ltd., Shanghai, China) series (75%, 80%, 90%, 95%, and 100%), dried using a critical point dryer (Quorum K850, Quorum Technologies Ltd., Laughton, East Sussex, UK), sputter-coated with gold using a Hitachi MC1000 ion sputter coater (Hitachi High-Tech Corporation, Tokyo, Japan), and examined using a Hitachi SU8100 field-emission scanning electron microscope (Hitachi High-Tech Corporation, Tokyo, Japan) at an accelerating voltage of 3 kV.

The present study was designed as a descriptive ultrastructural comparison based on one SEM specimen per species. Accordingly, SEM observations were used to document and qualitatively compare the ultrastructural characteristics of transparent and opaque wing regions in the examined specimens, rather than to assess intraspecific variation or to make statistical inferences about species-level differences.

### 2.3. Morphological Comparison of Wing Scales

SEM images were used to compare scale morphology and ultrastructure between transparent and opaque wing regions within each species and between the two species. Characters examined included scale morphology and orientation, surface ornamentation, ridge and cross-rib organization, perforation patterns, and internal structures, including laminae and trabeculae. These characters were recorded and comparatively assessed to identify structural differences between transparent and opaque wing regions and to determine whether similar transparent wing phenotypes in the two species were associated with similar or distinct scale-level modifications. Because only one individual per species was examined, the observed morphological differences were interpreted as descriptive observations of the examined specimens and were not used to assess intraspecific variation or to make statistical inferences about species-level differences. The SEM observations were used for qualitative morphological comparison rather than quantitative assessment. Structural characters were documented descriptively, and no statistical analyses were performed. SEM images were processed with Adobe Photoshop CC (Adobe Inc., San Jose, CA, USA).

## 3. Results

### 3.1. Morphology and Distribution of Transparent Wing Regions

Both *A*. *emma* and *T*. *penangae* possess conspicuous transparent regions on the forewings, but the distribution and configuration of these regions differ between the two species. In *A*. *emma*, several transparent windows are present on the forewing, including a relatively large transparent window in the basal region (Figure 2A,B). In contrast, the basal region of the forewing in *T*. *penangae* bears a yellow longitudinal stripe rather than a transparent window, while the transparent windows are distributed more distally on the wing (Figure 3A,B). Differences are also evident in the hindwings: the basal portion of the hindwing is transparent in *A*. *emma*, whereas the corresponding region in *T*. *penangae* is covered with yellow scales.

### 3.2. Wing-Scale Ultrastructure in A. emma

#### 3.2.1. Scales from Transparent Wing Regions

SEM examination of the transparent wing region of the examined *A*. *emma* specimen showed limited coverage, with substantial areas of the underlying wing membrane exposed between scales (Figure 4A,B). Individual scales were elongated, narrow, and needle-like, gradually tapering toward a pointed distal end, with a narrow basal stalk inserted into the scale socket (Figure 4C). At higher magnification, the scale surface exhibited regularly arranged longitudinal ridges interconnected by closely spaced transverse cross-ribs, forming a lattice-like pattern (Figure 4D). Conspicuous perforations such as those observed in scales from the opaque wing region were not evident in the examined scales from the transparent region.

#### 3.2.2. Scales from Opaque Wing Regions

In the opaque forewing region of the examined *A*. *emma* specimen, the wing membrane was covered by broad, leaf-shaped scales arranged in an overlapping, imbricate pattern (Figure 4E,F). Individual scales showed several shallow indentations along the distal margin and prominent longitudinal ridges extending along the scale surface (Figure 4G). At higher magnification, adjacent longitudinal ridges were interconnected by numerous transverse cross-ribs, and conspicuous circular to irregular perforations occurred within the inter-ridge spaces, producing a reticulate surface architecture (Figure 4H).

#### 3.2.3. Internal Architecture of Scales from Transparent and Opaque Wing Regions

Longitudinal views obtained by SEM revealed that scales from the transparent and opaque wing regions of the examined *A*. *emma* specimen possessed a hollow internal architecture consisting of upper and lower laminae interconnected by internal trabeculae (Figure 5). In scales from the transparent wing region, the trabeculae were slender and occurred at fairly regular intervals between the upper and lower laminae (Figure 5A,B). In scales from the opaque wing region, the trabeculae also connected the upper and lower laminae (Figure 5C,D). Thus, the two region types shared the same basic internal organization but differed in the observed configuration of the internal trabecular supports.

### 3.3. Wing-Scale Ultrastructure in T. penangae

#### 3.3.1. Scales from Transparent Wing Regions

SEM observations of the transparent wing region of the examined *T. penangae* specimen showed substantial areas of exposed wing membrane between scales. The scales projected from the wing membrane rather than lying closely appressed to it (Figure 6A–C). Representative scales exhibited a bud-like morphology, consisting of a narrow basal stalk and an expanded distal portion formed by several folded lamellar lobes (Figure 6B,C). The distal lamellar surfaces exhibited prominent longitudinal ridges interconnected by regularly spaced transverse cross-ribs, forming a reticulate surface pattern (Figure 6D). Variation in distal morphology was also observed, with some scales showing more strongly folded or irregularly arranged lamellar lobes (Figure 6D and Figure S2A). An additional morphological variant showed a more compact configuration, with several elongated lamellar folds arranged approximately in parallel (Figure S2B).

#### 3.3.2. Scales from Opaque Wing Regions

In the opaque wing region of the *T. penangae* specimen, broad, leaf-shaped scales covered the wing surface in an overlapping, imbricate arrangement (Figure 6E,F). Individual scales exhibited several distinct indentations along the distal margin and prominent, parallel longitudinal ridges extending along the scale surface (Figure 6F,G). At higher magnification, numerous oval to irregular perforations were visible between adjacent longitudinal ridges, which were interconnected by transverse cross-ribs to form a reticulate surface pattern (Figure 6G,H).

#### 3.3.3. Internal Architecture of Scales from Transparent and Opaque Wing Regions

SEM observations of longitudinally fractured scales revealed that scales from both the transparent and opaque wing regions of the examined *T. penangae* specimen possessed a hollow internal architecture, with upper and lower laminae interconnected by trabeculae (Figure 7). In scales from the transparent wing region, slender trabeculae extended between the upper and lower laminae and formed a pillar-like internal framework spanning the scale cavity (Figure 7A,B). In scales from the opaque wing region, trabeculae similarly interconnected the upper and lower laminae, with prominent pillar-like and locally expanded supports visible within the internal cavity (Figure 7C,D).

### 3.4. Interspecific Comparison of Wing-Scale Ultrastructure

Comparative SEM observations revealed both shared and species-specific features in the wing-scale ultrastructure of *A. emma* and *T. penangae*. In the transparent forewing regions, substantial areas of wing membrane remained exposed between scales in both specimens. In the opaque regions, broad, leaf-shaped scales formed overlapping arrangements over the wing membrane. Opaque-region scales of both specimens possessed prominent longitudinal ridges interconnected by transverse cross-ribs, with conspicuous perforations between adjacent ridges, although their overall outlines and distal margins differed.

More pronounced interspecific differences were observed in scales from the transparent wing regions. In *A. emma*, these scales were elongated, narrow, and needle-like, with tapered distal ends. In *T. penangae*, the scales projected from the wing membrane and exhibited a distinctive bud-like morphology, consisting of a narrow basal stalk and an expanded distal portion formed by folded lamellar lobes. Variation in the arrangement and degree of folding of these distal lamellar structures was also observed in *T. penangae*. Conspicuous perforations comparable to those of the opaque-region scales were not evident in the examined transparent-region scales of either species.

Longitudinal SEM observations showed that scales from both wing regions in the two species shared a similar basic internal organization, consisting of upper and lower laminae interconnected by trabeculae. In both species, trabeculae in scales from the transparent wing regions generally appeared relatively slender, whereas those in scales from the opaque wing regions appeared broader and more robust. Thus, the principal interspecific differences were most evident in the external morphology and three-dimensional configuration of scales associated with the transparent wing regions, whereas the basic internal scale organization was similar in the two species.

## 4. Discussion

### 4.1. Different Structural Strategies Underlying Similar Wing Transparency

Wing transparency has evolved repeatedly across Lepidoptera and is associated with substantial diversity in scale density, morphology, orientation, pigmentation, and micro- and nanostructural organization rather than a single structural solution [5,9,11,23]. The present study adds to this diversity by showing that the macroscopically similar transparent wing regions of *A. emma* and *T. penangae* are associated with markedly different modifications of wing scales.

In the examined specimens, transparent wing regions displayed exposed areas of the wing membrane between scales; however, scale morphology and arrangement differed between the two specimens. In the *A. emma* specimen possessed scales in the transparent region were elongated, narrow, and needle-like. In contrast, scales in the transparent region of the *T. penangae* specimen projected from the wing membrane and exhibited three-dimensionally folded, bud-like distal structures. These contrasting configurations may represent different structural routes by which the area of wing membrane covered by scales may be limited. However, because scale density, dimensions, orientation, and projected area were not quantified, the relative contribution of these features to wing transparency could not be determined from the present observations.

These observations suggest that, in the two species examined, the structural modifications of transparent-region scales are not solely attributable to simple structural reduction, because the basic upper- and lower-lamina organization interconnected by trabeculae is retained. Instead, the marked differences in external scale morphology appear to represent different modifications of a broadly shared internal structural framework. This pattern is consistent with broader comparative studies showing that similar transparent wing phenotypes can arise through different combinations of scale reduction, altered orientation, modified scale geometry, and changes in wing-surface architecture [5,11,23]. The contrasting scale configurations observed here therefore further illustrate the structural flexibility through which similar transparent wing appearances can be achieved in Lepidoptera.

Although the scale modifications observed here may influence light transmission through changes in scale coverage, morphology, and orientation, their optical effects were not directly examined in the present study. Previous studies of transparent Lepidoptera have shown that reduced scale density, modified scale morphology, and upright or erected scale orientation can be associated with increased exposure of the wing membrane and higher light transmission [5,23,24]. Comparative analyses have further demonstrated that light transmittance can vary with scale type and density, indicating that multiple structural features may contribute jointly to the degree of wing transparency [16]. However, the relative contribution of these factors is likely to differ among species and structural configurations. Quantitative measurements of scale density and orientation, combined with optical characterization, will therefore be required to determine how the contrasting scale architectures of *A. emma* and *T. penangae* contribute to their similar transparent wing appearance.

### 4.2. Transparency-Associated Scale Modifications in a Broader Lepidopteran Context

Structural modifications of wing scales associated with transparency have been reported in several distantly related lepidopteran taxa. In the glasswing butterfly *Greta oto*, transparent wing regions contain a reduced density of highly modified bristle-like and narrow forked scales, in contrast to the larger, flattened scales of adjacent opaque regions [5]. A comparable modification has also been reported in the saturniid moth *Rothschildia lebeau*, in which the transparent wing “windows” are sparsely covered by narrow, hair-like bristles that project nearly perpendicular to the wing surface [18]. Similarly, transparency in *Chorinea sylphina* has been associated with sparse and upright scales [24]. These published examples provide useful comparative context for the elongated, narrow scales and exposed wing membrane observed in the transparent region of the examined *A. emma* specimen.

Other transparent-wing Lepidoptera demonstrate that extensive reduction in scale shape is not always required. In *Phanus vitreus*, for example, transparency is associated with reduced pigmentation and a nearly vertical orientation of otherwise relatively conventional paddle-like scales [16]. The scales observed in the transparent region of the examined *T. penangae* specimen likewise projected away from the wing membrane, but their expanded and folded bud-like morphology represents a different structural configuration. Taken together with broader comparative surveys of transparent Lepidoptera, these examples indicate that reduced scale coverage, scale narrowing, modified orientation, and specialized three-dimensional scale morphology can occur in different combinations in association with wing transparency [11].

The structures observed in *A. emma* and *T. penangae* therefore indicate that there is no single generalized scale morphology associated with transparent wing regions. Rather, they add two contrasting configurations to the known structural diversity of transparency-associated scales in Lepidoptera. Whether these similarities among taxa reflect comparable functional solutions or different developmental and evolutionary processes cannot be determined from morphology alone and will require broader comparative and phylogenetic analyses.

### 4.3. Limitations and Future Directions

The present study was based on descriptive SEM observations of one adult individual per species. Consequently, the observed morphological patterns should be interpreted as descriptive features of the examined specimens rather than quantitative species-level differences. Variation associated with individual identity, sex, wing surface, wing position, population, or geographic origin could not be evaluated. In addition, individual scales from the same specimen do not constitute independent biological replicates; therefore, quantitative comparisons based on multiple scales from these two specimens alone could lead to pseudoreplication. Future studies incorporating multiple independent individuals of both sexes should quantify scale dimensions, scale density, orientation, trabecular morphology, and associated variation to determine the consistency and extent of the structural patterns documented here.

In addition, the functional consequences of the observed scale modifications remain to be determined. Transparency in Lepidoptera is not solely an optical phenomenon, but may also be associated with broader ecological and physiological consequences. Previous studies have shown that wing transparency can influence visual concealment, while modifications associated with transparent wings may also involve trade-offs in thermoregulation and hydrophobic performance [7,13,25,26,27]. Future work combining quantitative morphology with optical, thermal, and functional measurements would therefore help determine whether the contrasting scale configurations of *A. emma* and *T. penangae* differ not only in their contribution to light transmission but also in other aspects of wing performance.

Finally, the developmental and evolutionary origins of these contrasting scale configurations remain unresolved. Recent studies have begun to reveal the cellular and physical processes underlying butterfly scale morphogenesis, including mechanisms involved in ridge formation, while broader developmental studies highlight the genetic and cellular basis of insect cuticular micro- and nanostructures [28,29]. Comparative developmental analyses could therefore help determine how the slender needle-like scales of *A. emma* and the folded bud-like scales of *T. penangae* arise from the basic lepidopteran scale developmental program. Combined with broader taxonomic and phylogenetic sampling, such approaches could further clarify whether these configurations represent lineage-specific modifications or more widespread structural strategies associated with transparent wings [3,11].

## 5. Conclusions

SEM observations revealed distinct wing-scale modifications associated with the transparent wing regions of *A*. *emma* and *T*. *penangae*. In *A. emma*, transparent-region scales were elongated, narrow, and needle-like, with substantial areas of wing membrane exposed between scales. In *T. penangae*, transparent-region scales also left substantial areas of wing membrane exposed but exhibited a distinctive bud-like morphology with folded distal lamellar structures and projected from the wing membrane. In the opaque regions of both specimens, broad, leaf-shaped scales formed overlapping arrangements and possessed prominent longitudinal ridges, transverse cross-ribs, and conspicuous perforations.

These observations show that macroscopically similar transparent wing regions can be associated with different scale-level structural modifications while retaining a common underlying scale architecture. Because only one male individual of each species was examined, these patterns should not be interpreted as quantitative species-level differences. The study provides a descriptive basis for future work using multiple independent specimens together with quantitative morphology, optical measurements, and ecological or evolutionary analyses.

## Figures and Tables

**Figure 1:**
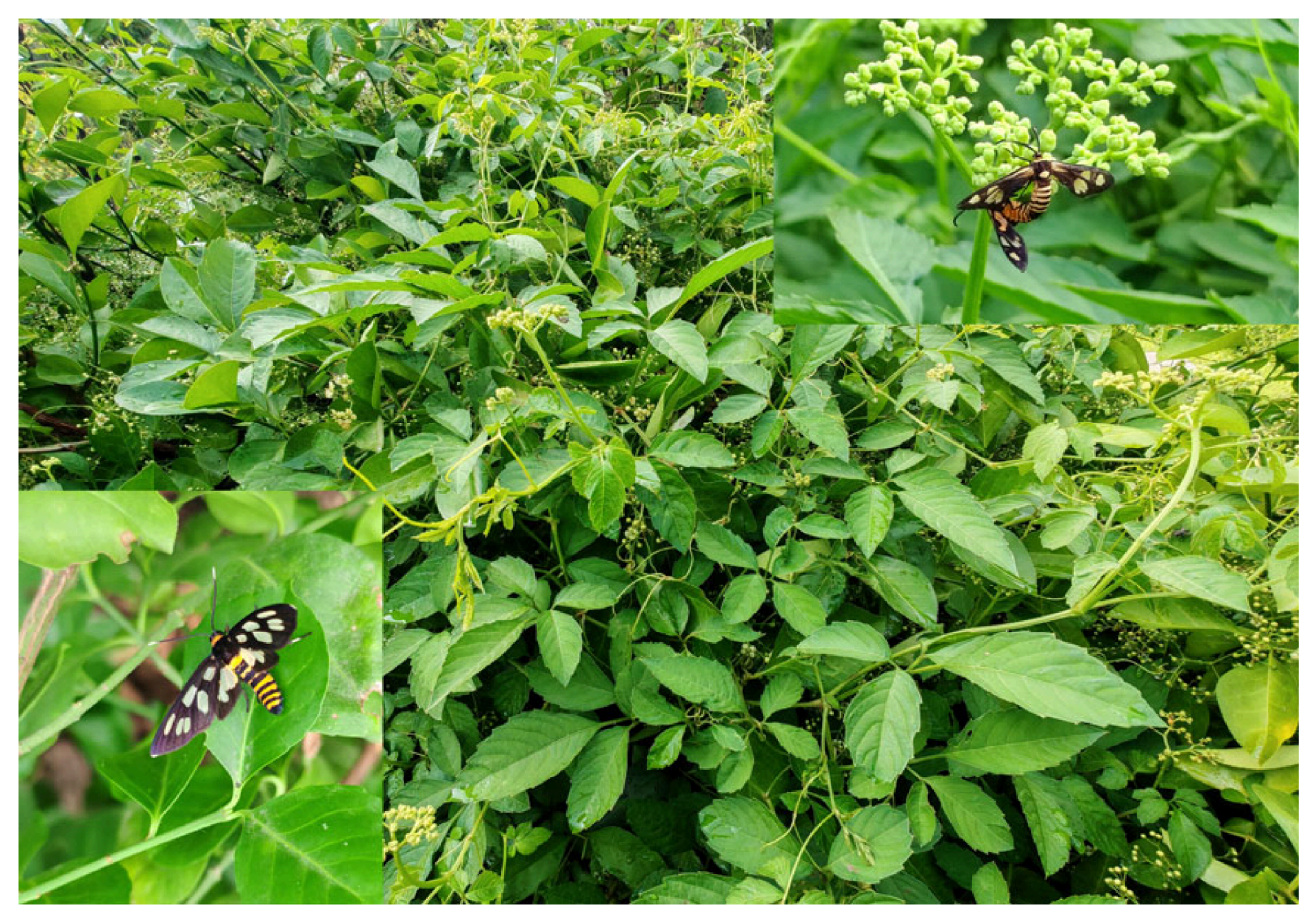
The common host plant, *Cayratia japonica* (Thunb.), and the two externally similar moth species examined in this study. *Amata emma* (Butler) (lower left) and *Thyrassia penangae* Moore (upper right) are both associated with *C*. *japonica*. All photographs were taken by the authors at Zhengzhou University, Zhengzhou, China.

**Figure 2:**
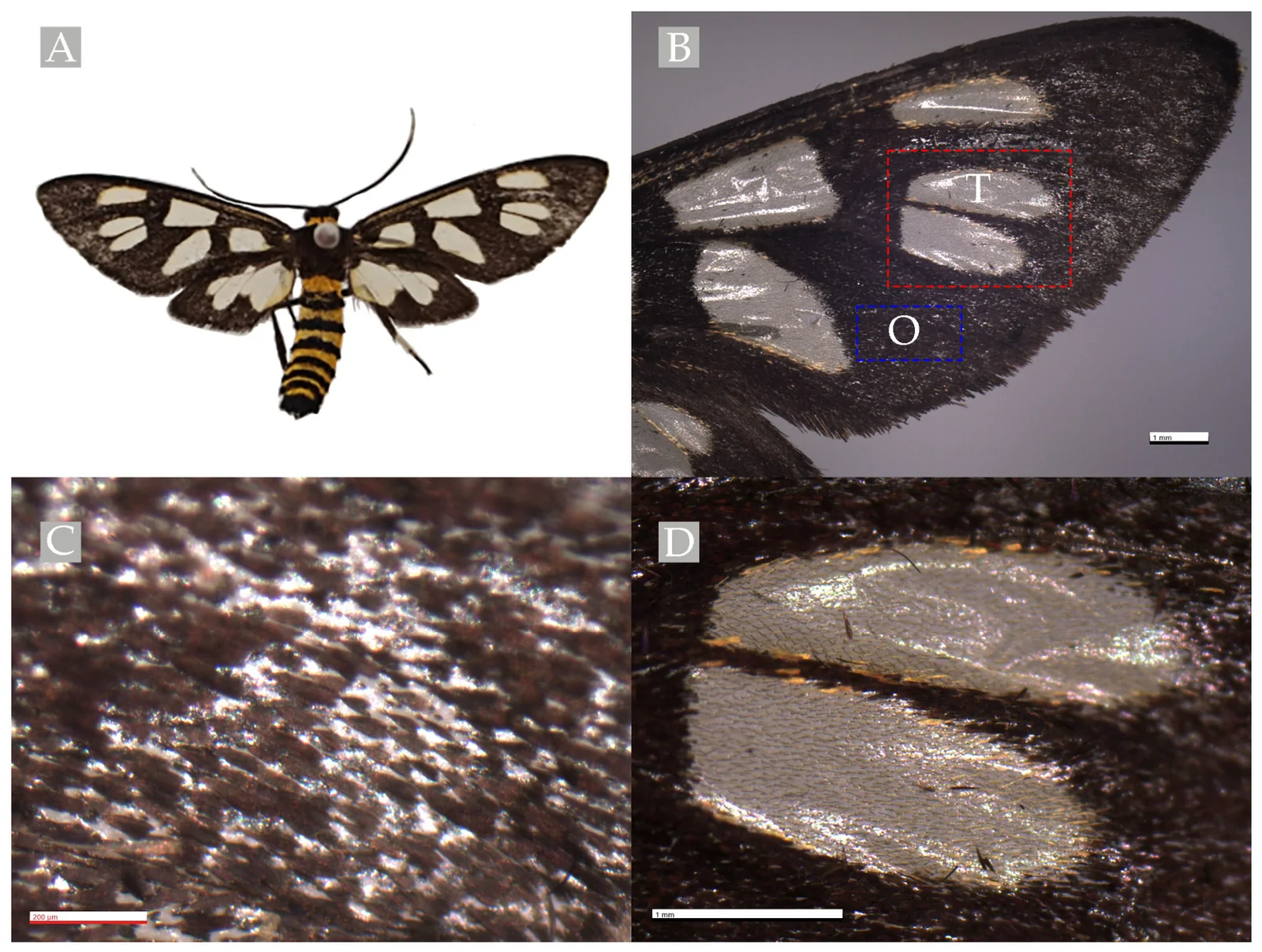
External morphology of *A. emma*. (**A**) Adult specimen. (**B**) Forewing showing the transparent wing window and the locations selected for SEM sampling. The red box labeled “T” indicates the sampled transparent region, and the blue box labeled “O” indicates the sampled adjacent opaque region covered with dark scales. (**C**) Enlarged view of the opaque wing region. (**D**) Enlarged view of the transparent wing region.

**Figure 3:**
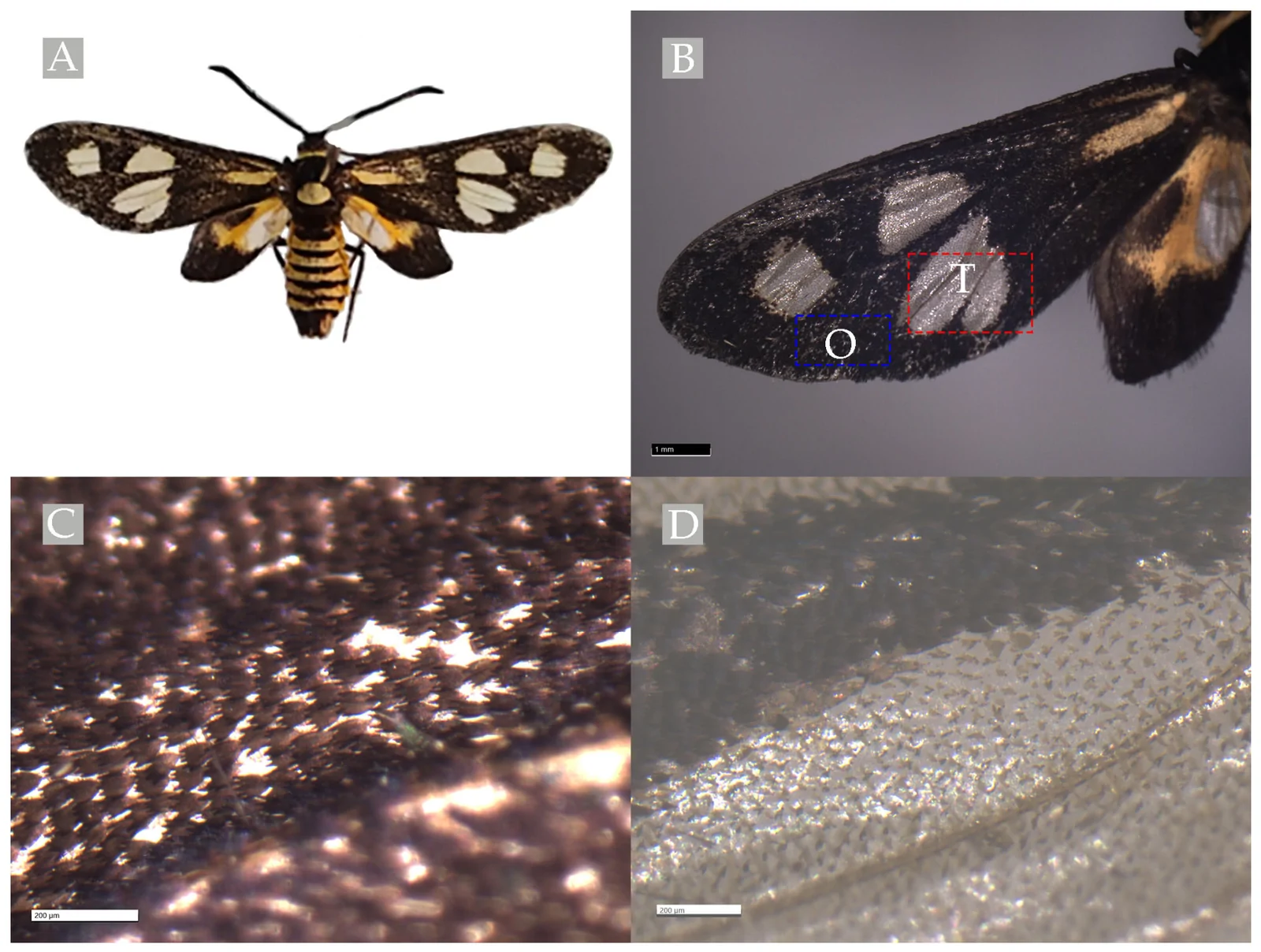
External morphology of *T. penangae*. (**A**) Adult specimen. (**B**) Forewing showing the transparent wing window and the locations selected for SEM sampling. The red box labeled “T” indicates the sampled transparent region, and the blue box labeled “O” indicates the sampled adjacent opaque region covered with dark scales. (**C**) Enlarged view of the opaque wing region. (**D**) Enlarged view of the transparent wing region.

**Figure 4:**
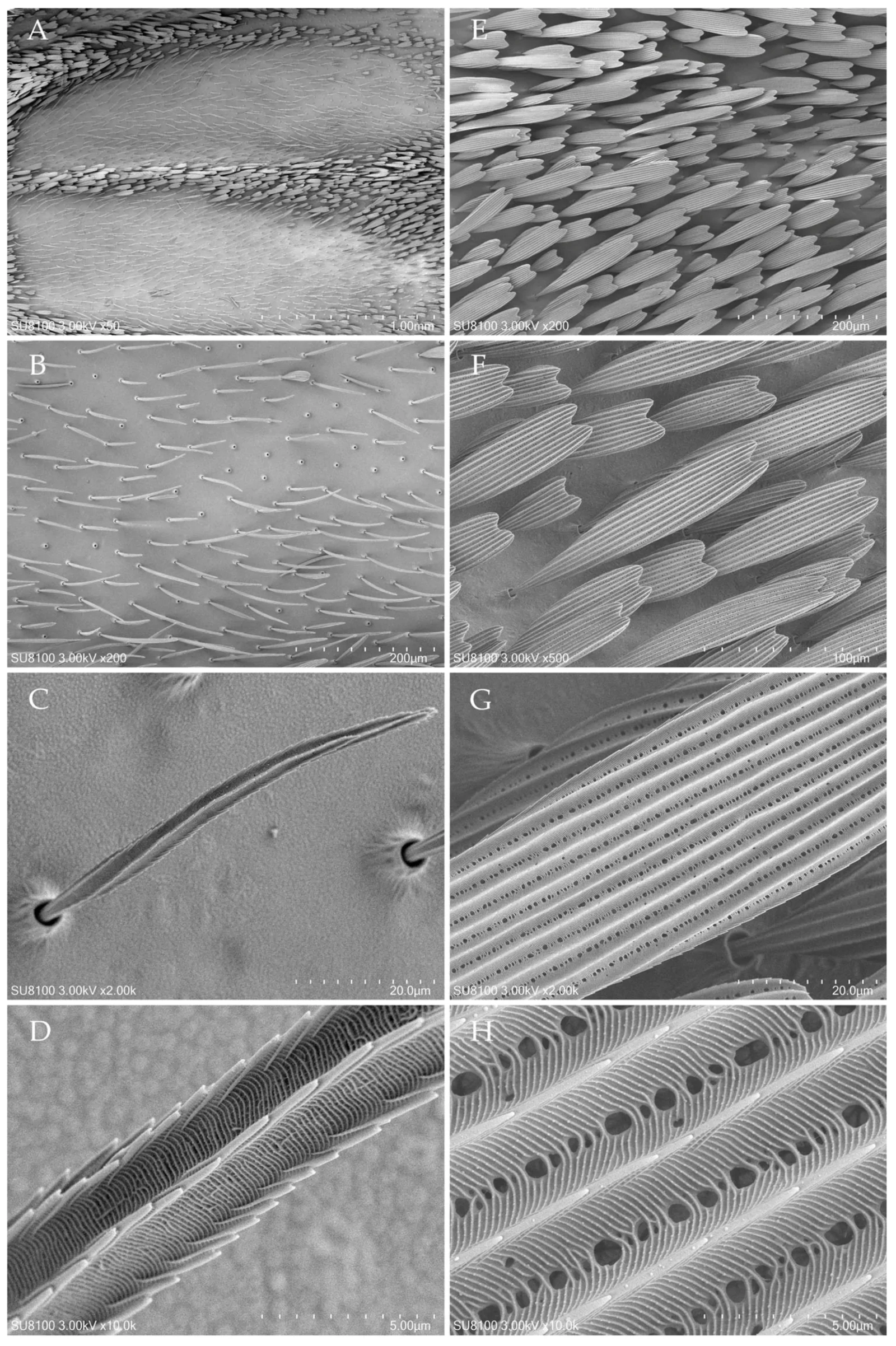
Ultrastructural characteristics of scales from transparent and opaque wing regions of *A*. *emma*. (**A**–**D**) Scales from transparent wing regions. (**A**) General view showing sparse scale coverage in the transparent wing region. (**B**) Sparse arrangement of slender scales on the wing membrane. (**C**) Individual needle-like scale showing the tapered distal end and narrow basal stalk. (**D**) High-magnification view of the scale surface showing longitudinal ridges interconnected by transverse cross-ribs. (**E**–**H**) Scales from the opaque wing region. (**E**) General view showing dense scale coverage in the opaque wing region. (**F**) Overlapping arrangement of broad scales on the wing membrane. (**G**) Individual leaf-shaped scale showing shallow serrations along the distal margin. (**H**) High-magnification view of the scale surface showing longitudinal ridges, transverse cross-ribs, and numerous perforations between adjacent ridges. Scale bars as indicated.

**Figure 5:**
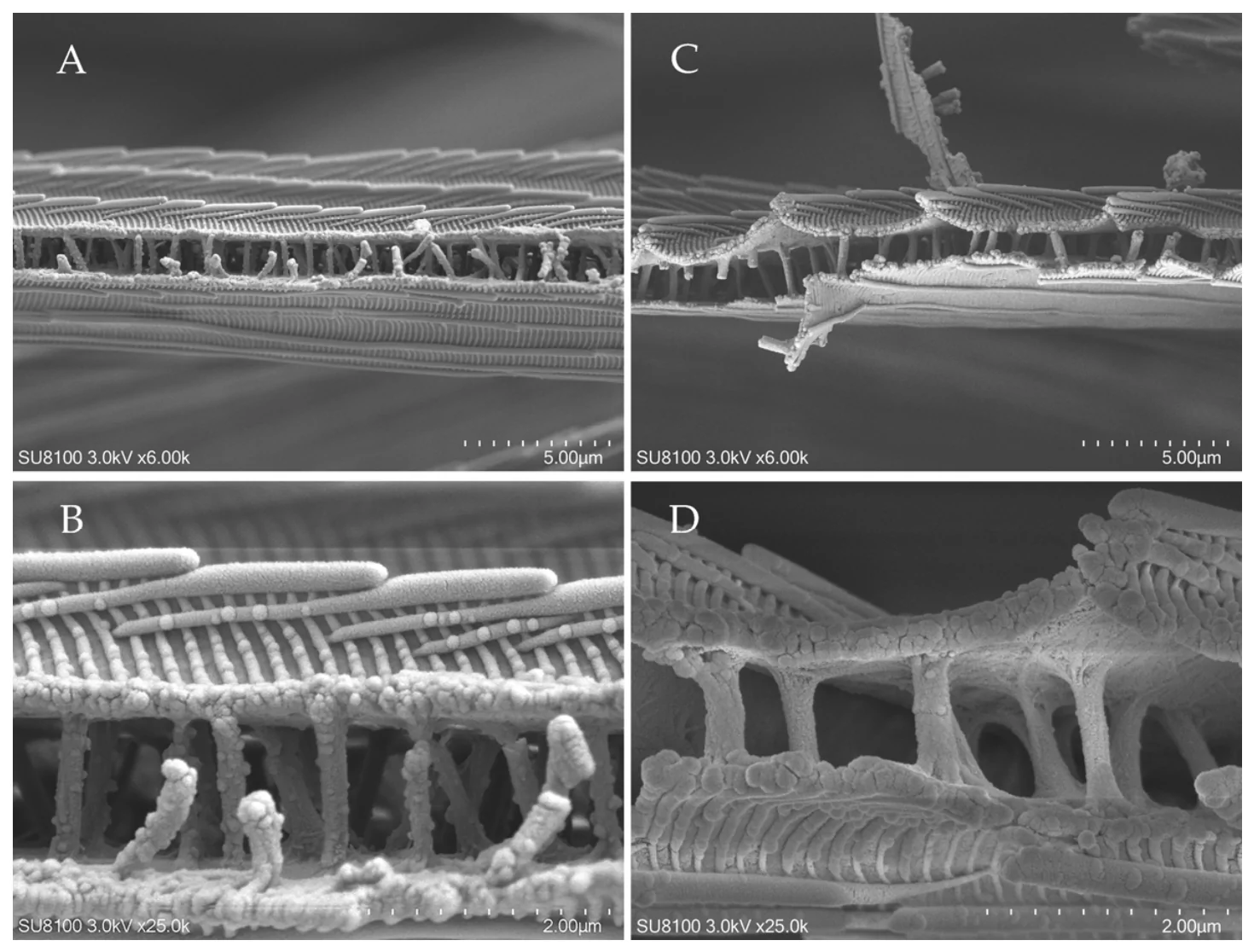
Internal ultrastructure of scales from the transparent and opaque wing regions of *A*. *emma*. (**A**,**B**) Longitudinal views of scales from the transparent wing region showing the upper and lower laminae interconnected by relatively slender trabeculae. (**C**,**D**) Longitudinal views of scales from the opaque wing region showing the upper and lower laminae interconnected by broader, more robust trabeculae. Scale bars as indicated.

**Figure 6:**
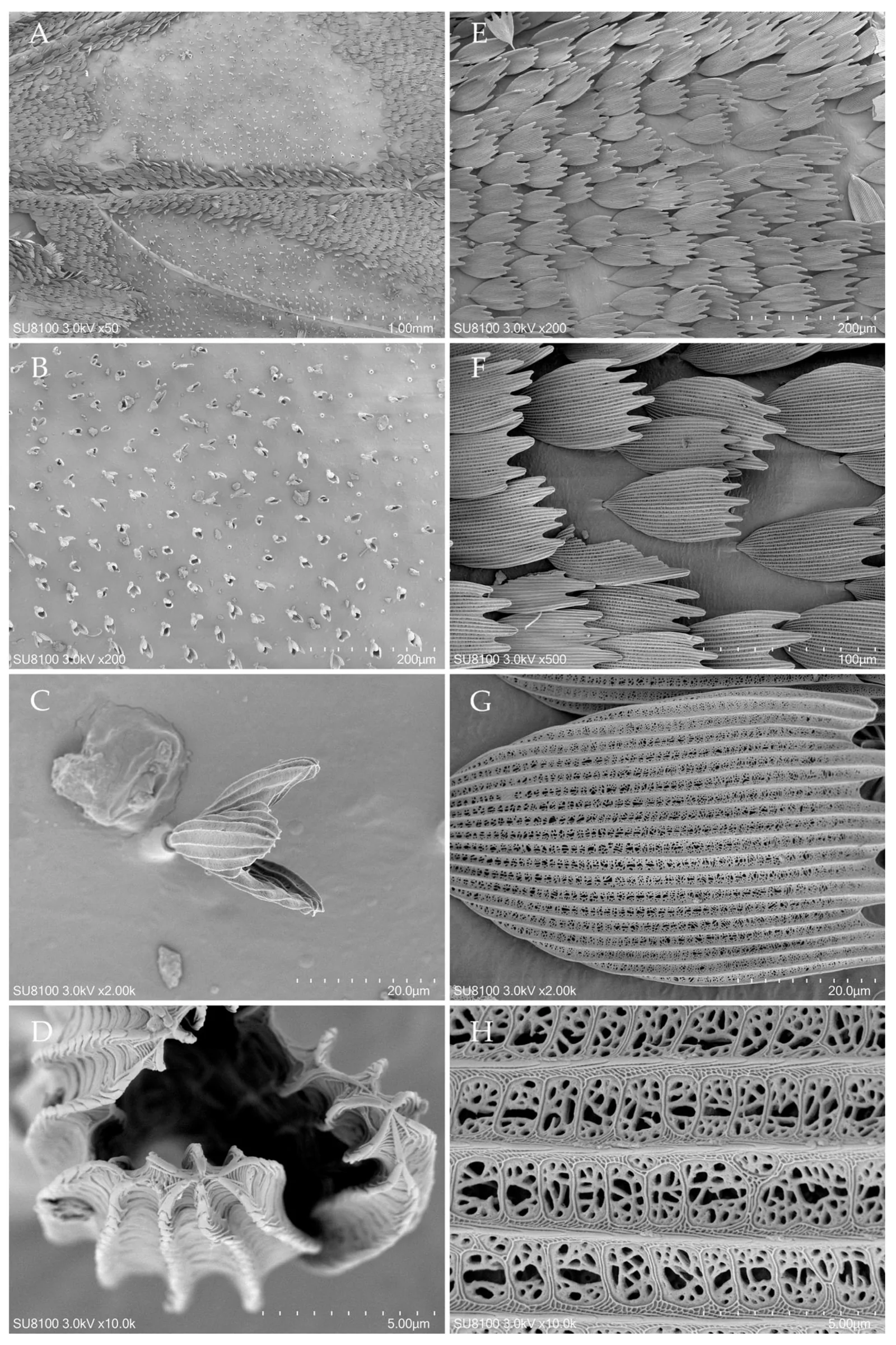
Ultrastructural characteristics of scales from the transparent and opaque wing regions of *T. penangae*. (**A**–**D**) Scales from the transparent wing region. (**A**) General view showing sparse scale distribution in the transparent wing region. (**B**) Enlarged view of scales projecting from the wing membrane. (**C**) Individual bud-like scale showing the narrow basal stalk and expanded distal portion. (**D**) High-magnification view of the distal lamellar surface showing longitudinal ridges interconnected by transverse cross-ribs. (**E**–**H**) Scales from the opaque wing region. (**E**) General view showing dense, overlapping scale coverage in the opaque wing region. (**F**) Enlarged view of broad, leaf-shaped scales showing indentations along the distal margin. (**G**) Scale surface showing parallel longitudinal ridges and perforations between adjacent ridges. (**H**) High-magnification view showing the perforation pattern and ridge–cross-rib organization. Scale bars as indicated.

**Figure 7:**
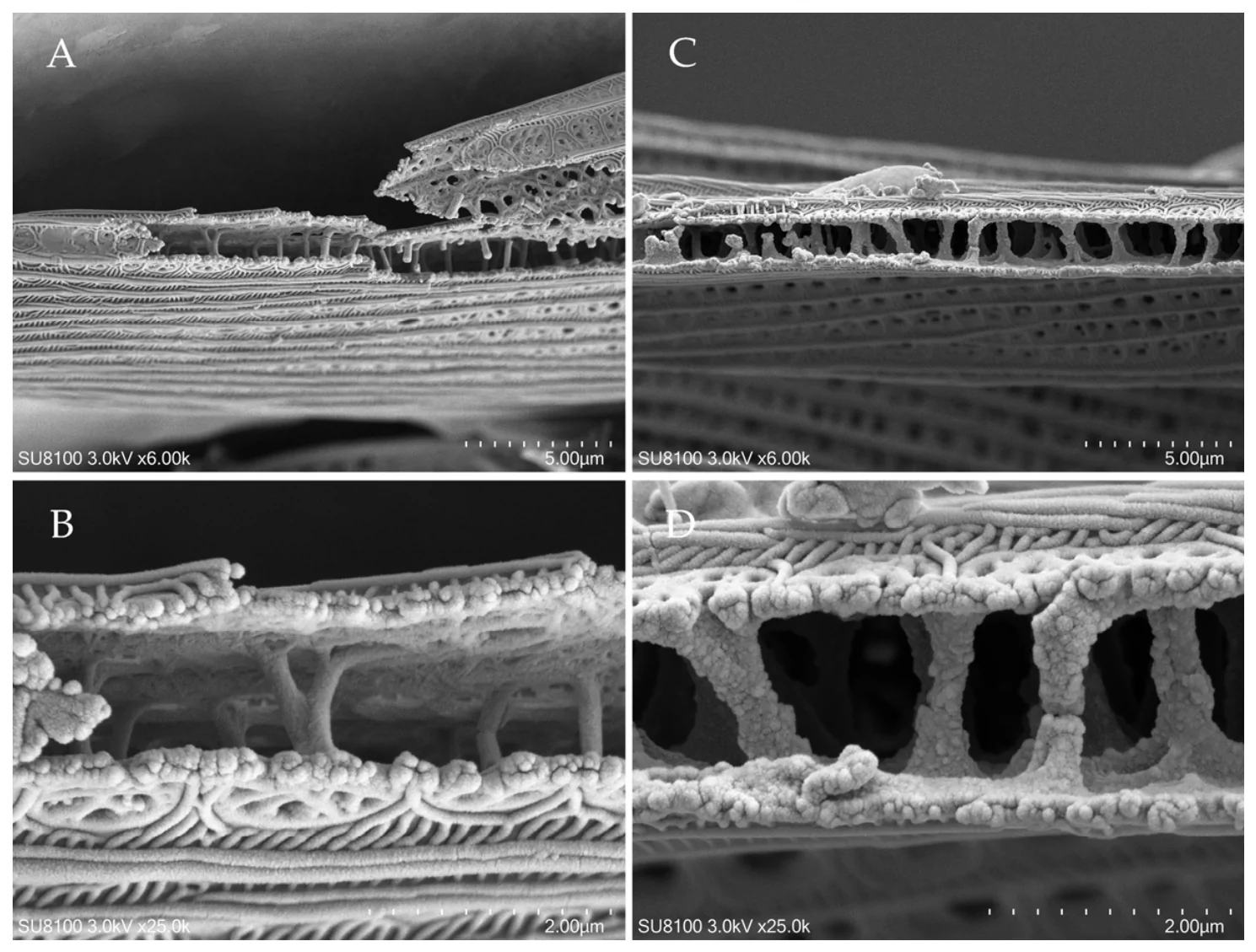
Internal ultrastructure of scales from the transparent and opaque wing regions of *T. penangae*. (**A**,**B**) Longitudinal views of scales from the transparent wing region showing the upper and lower laminae interconnected by relatively slender trabeculae. (**C**,**D**) Longitudinal views of scales from the opaque wing region showing the upper and lower laminae interconnected by broader, more robust trabeculae. Scale bars as indicated.

## Data Availability

The original high-resolution SEM micrographs underlying the images presented in the main figures, together with associated metadata, are provided in the Supplementary Materials. Additional original SEM micrographs generated during this study are available from the corresponding author upon reasonable request.

## Supplementary Materials

The following supporting information can be downloaded at https://www.mdpi.com/article/10.3390/insects17100999/s1. Figure S1: Male genitalia of the two examined moth specimens used to support species identification; Figure S2: Morphological variation in scales from the transparent wing region of *T. penangae*; Table S1: Metadata and correspondence between the original SEM micrographs and the SEM images presented in the main figures.
