# Supplementary material for: Comparative Wing-Scale Ultrastructure Reveals Distinct Structural Modifications Associated with Transparent Wing Regions in Two Moth Species (Lepidoptera)
Source: Insects. 2026 Sep 27;17(10):999. doi: 10.3390/insects17100999 (PMC13705125; doi:10.3390/insects17100999)
Supplement: Supplementary file 1 [file insects-17-00999-s001.zip › insects-4490699-supplementary.pdf]

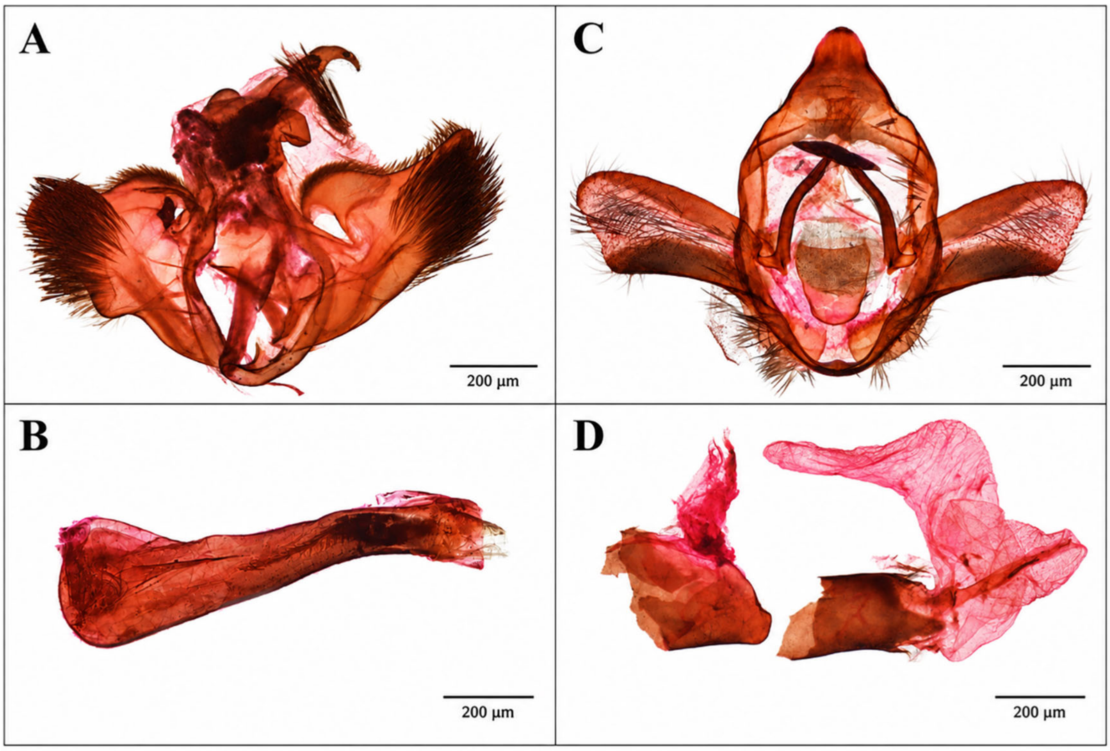

Figure S1. Male genitalia of the specimens used for species identification. (A, B) *Amata emma*: (A) male genitalia; (B) aedeagus. (C, D) *Thyrassia penangae*: (C) male genitalia; (D) aedeagus. The genital morphology was consistent with the diagnostic characteristics described by Wu [22]. Scale bars as indicated.

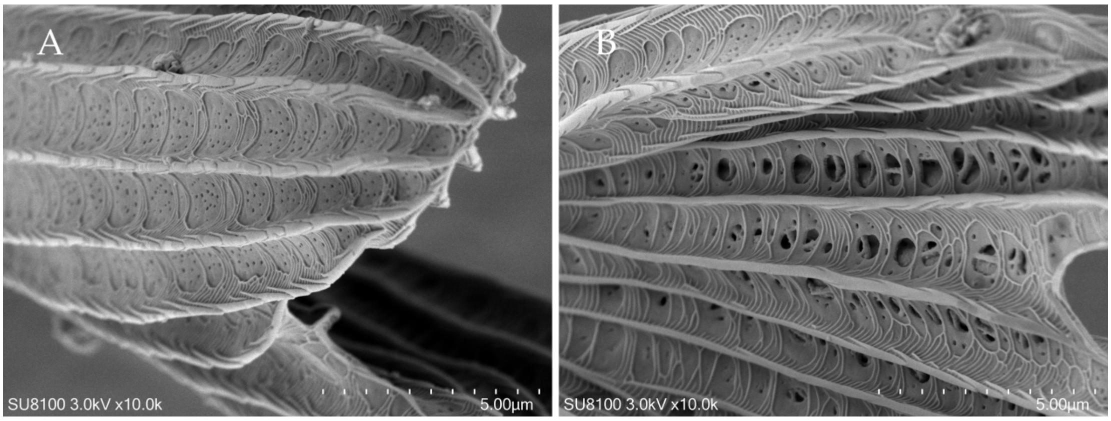

Figure S2. Variation in scale morphology observed within the transparent wing region of the examined *T. penangae* specimen. (A) Scale exhibiting strongly folded and irregularly arranged distal lamellar lobes. (B) Scale exhibiting a more compact configuration, with elongated lamellar folds arranged approximately in parallel. Scale bars as indicated.

Table S1 Metadata and correspondence of the original SEM micrographs underlying the images presented in the main and supplementary figures.

**Original file**	**Species**	**Wing region**	**Structure examined**	**Corresponding figure panel**
Fig4A_*A.emma*_Transparent_GeneralView.tif	*A. emma*	Transparent	General view of wing scales	Fig. 4A
Fig4B_*A.emma*_Transparent_ScaleArrangement.tif	*A. emma*	Transparent	Scale arrangement on wing membrane	Fig. 4B
Fig4C_*A.emma*_Transparent_NeedleLikeScale.tif	*A. emma*	Transparent	Individual needle-like scale	Fig. 4C
Fig4D_*A.emma*_Transparent_ScaleSurface.tif	*A. emma*	Transparent	Scale surface, ridges and cross-ribs	Fig. 4D
Fig4E_*A.emma*_Opaque_GeneralView.tif	*A. emma*	Opaque	General view of wing scales	Fig. 4E
Fig4F_*A.emma*_Opaque_ScaleArrangement.tif	*A. emma*	Opaque	Overlapping scale arrangement	Fig. 4F
Fig4G_*A.emma*_Opaque_LeafShapedScale.tif	*A. emma*	Opaque	Individual leaf-shaped scale	Fig. 4G
Fig4H_*A.emma*_Opaque_ScaleSurface.tif	*A. emma*	Opaque	Scale surface, ridges, cross-ribs, and perforations	Fig. 4H
Fig5A_*A.emma*_Transparent_InternalStructure.tif	*A. emma*	Transparent	Longitudinal view of scale internal structure	Fig. 5A
Fig5B_*A.emma*_Transparent_InternalStructure.tif	*A. emma*	Transparent	Longitudinal view of scale internal structure	Fig. 5B
Fig5C_*A.emma*_Opaque_InternalStructure.tif	*A. emma*	Opaque	Longitudinal view of scale internal structure	Fig. 5C

Original file	Species	Wing region	Structure examined	Corresponding figure panel
Fig5D_*A.emma*_Opaque_InternalStructure.tif	*A. emma*	Opaque	Longitudinal view of scale internal structure	Fig. 5D
Fig6A_*T.penangae*_Transparent_GeneralView.tif	*T. penangae*	Transparent	General view of wing scales	Fig. 6A
Fig6B_*T.penangae*_Transparent_ProjectingScales.tif	*T. penangae*	Transparent	Scales projecting from wing membrane	Fig. 6B
Fig6C_*T.penangae*_Transparent_BudLikeScale.tif	*T. penangae*	Transparent	Individual bud-like scale	Fig. 6C
Fig6D_*T.penangae*_Transparent_DistalSurface.tif	*T. penangae*	Transparent	Distal lamellar surface, ridges and cross-ribs	Fig. 6D
Fig6E_*T.penangae*_Opaque_GeneralView.tif	*T. penangae*	Opaque	General view of wing scales	Fig. 6E
Fig6F_*T.penangae*_Opaque_LeafShapedScales.tif	*T. penangae*	Opaque	Broad leaf-shaped scales	Fig. 6F
Fig6G_*T.penangae*_Opaque_ScaleSurface.tif	*T. penangae*	Opaque	Scale surface, ridges and perforations	Fig. 6G
Fig6H_*T.penangae*_Opaque_SurfaceDetail.tif	*T. penangae*	Opaque	Ridge–cross-rib organization and perforations	Fig. 6H
Fig7A_*T.penangae*_Transparent_InternalStructure.tif	*T. penangae*	Transparent	Longitudinal view of scale internal structure	Fig. 7A
Fig7B_*T.penangae*_Transparent_InternalStructure.tif	*T. penangae*	Transparent	Longitudinal view of scale internal structure	Fig. 7B
Fig7C_*T.penangae*_Opaq	*T.*	Opaque	Longitudinal view of	Fig. 7C

Original file	Species	Wing region	Structure examined	Corresponding figure panel
ue_InternalStructure.tif	*penangae*		scale internal structure	
Fig7D_*T.penangae*_Opaque_InternalStructure.tif	*T. penangae*	Opaque	Longitudinal view of scale internal structure	Fig. 7D
FigS2A_*T.penangae*_Transparent_FoldedLamellae.tif	*T. penangae*	Transparent	Scale with folded distal lamellar lobes	Fig. S2A
FigS2B_*T.penangae*_Transparent_CompactLamellae.tif	*T. penangae*	Transparent	Scale with compact distal lamellar configuration	Fig. S2B
